# On Discovering Thymus–Marrow Synergism

**DOI:** 10.3389/fimmu.2014.00588

**Published:** 2014-11-21

**Authors:** Henry N. Claman

**Affiliations:** ^1^Department of Medicine, University of Colorado School of Medicine, Denver, CO, USA; ^2^Department of Immunology, University of Colorado School of Medicine, Denver, CO, USA

**Keywords:** thymus, bone marrow, sheep red blood cells, stem cells, thymus–marrow synergism

In the 1960s, the thymus was an organ of mystery. Although it was full of lymphocytes, they made no antibodies. Furthermore, thymectomy (in mature animals) failed to produce immunological inadequacy. This situation changed when J.F.A.P. Miller did *neonatal thymectomy*, which was indeed followed by a syndrome including crippling of the immune response (1–3).

The possible role of the thymus was the focus of a several-day symposium organized by Robert A. Good in November, 1962 in Minneapolis. Its proceedings, *The Thymus in Immunobiology*, summarized the clinical and experimental data (4).

This mystery organ intrigued me. Systemically, immunized animals did not make antibody in the thymus as they did in lymph nodes or spleen. Maybe, we thought, there was some kind of blood–thymus barrier, which prevented systemic antigen from interacting with thymocytes in the thymic parenchyma. We used adoptive transfer of syngeneic cells into irradiated recipients. In this model, spleen cell suspensions responded to sheep erythrocyte (sheep red blood cells, SRBC) antigens by making hemolytic antibody in the recipient spleens and serum. Would thymus cell suspensions similarly prepared (so as to break any blood–thymus barrier) do the same?

I was a young investigator working in the late David W. Talmage’s lab at the University of Colorado Medical School in Denver. It was a stimulating environment. Edward A. Chaperon and R. Faser Triplett were post-doctoral fellows. On day 0, the mice were irradiated and then injected i.v. with spleen cells or thymus cells. On day 1, we injected the SRBC antigen IV. On day 5, we sacrificed the mice and looked for anti-SRBC-producing cells in the recipient spleens. The results were clear-cut. Recipients of donor spleen cells made many antibody-producing cells while recipients of donor thymus cells did not. Perhaps, we thought, 4-days of exposure to antigen after transfer might have been sufficient to get the mature spleen cells to make antibody but insufficient for the (putatively) immature thymocytes to do the same. We needed to lengthen the protocol.

The next experiments were identical on days 0 and 1, but on day-4 recipients got a booster injection of SRBC antigen, and we planned to sacrifice on day 8. This worked well in the group that received spleen cells but the recipients of thymus cells (the test group) were all dead by day 8. We figured that this represented radiation death in the thymus recipients, whereas the spleen recipients survived because of the hematopoietic stem cells in the inoculum.

We knew of the radio-protective effects of bone marrow cells, so it made sense to add an aliquot of such cells to the thymus inoculum. Indeed, the recipients of thymus-plus-bone marrow survived until day 8, but to our surprise, these recipients produced almost as much antibody as did spleen recipients. (Later we added a new group as another control, i.e., bone marrow cells only. They caused no significant antibody production.)

We called this phenomenon “thymus–marrow synergism,” and it was the first demonstration that two (presumably lymphoid) cell populations were needed for significant antibody production. We speculated that one sort of cell (the “effector”) made the antibody while another variety of cell from the other inoculum performed in an “auxiliary” mode. On the basis of indirect evidence, we postulated that the bone marrow provided the effector cells and the thymus cells were “auxiliary.” Support for this view had to await the definitive experiments by others.

Our experiments were published in 1966 in Proc. Soc. Exptl. Biol. Med. (5). The paper was widely acknowledged to have demonstrated cell–cell interaction in the antibody response. Additional findings by the three of us were considered important enough many years later when they were chosen as the first article to be mentioned in the *Journal of Immunology’s* new historical series, Pillars of Immunology.

This discovery was unexpected – almost representing serendipity. Not everyone was convinced. However, others used the paradigm to provide further elucidation of the mechanism of thymus–marrow synergism. Mitchell and Miller made great progress by identifying the antibody-forming cell as originating in the bone marrow (6). Additionally, Avrion Mitchison added the brilliant insight that *the carrier effect* was an example of T–B collaboration where the anti-hapten antibody was made by bone marrow-derived cells while the thymus-derived cells provided “help” (7–9).

## Conflict of Interest Statement

The author declares that the research was conducted in the absence of any commercial or financial relationships that could be construed as a potential conflict of interest.
